# Supplementary figures and images for: Dasatinib and quercetin senolytic treatment delays early onset intervertebral disc degeneration in SM/J mice
Source: Bone Res. 2026 Apr 14;14:42. doi: 10.1038/s41413-026-00526-4 (PMC13076796; doi:10.1038/s41413-026-00526-4)

Supplementary Figure 1

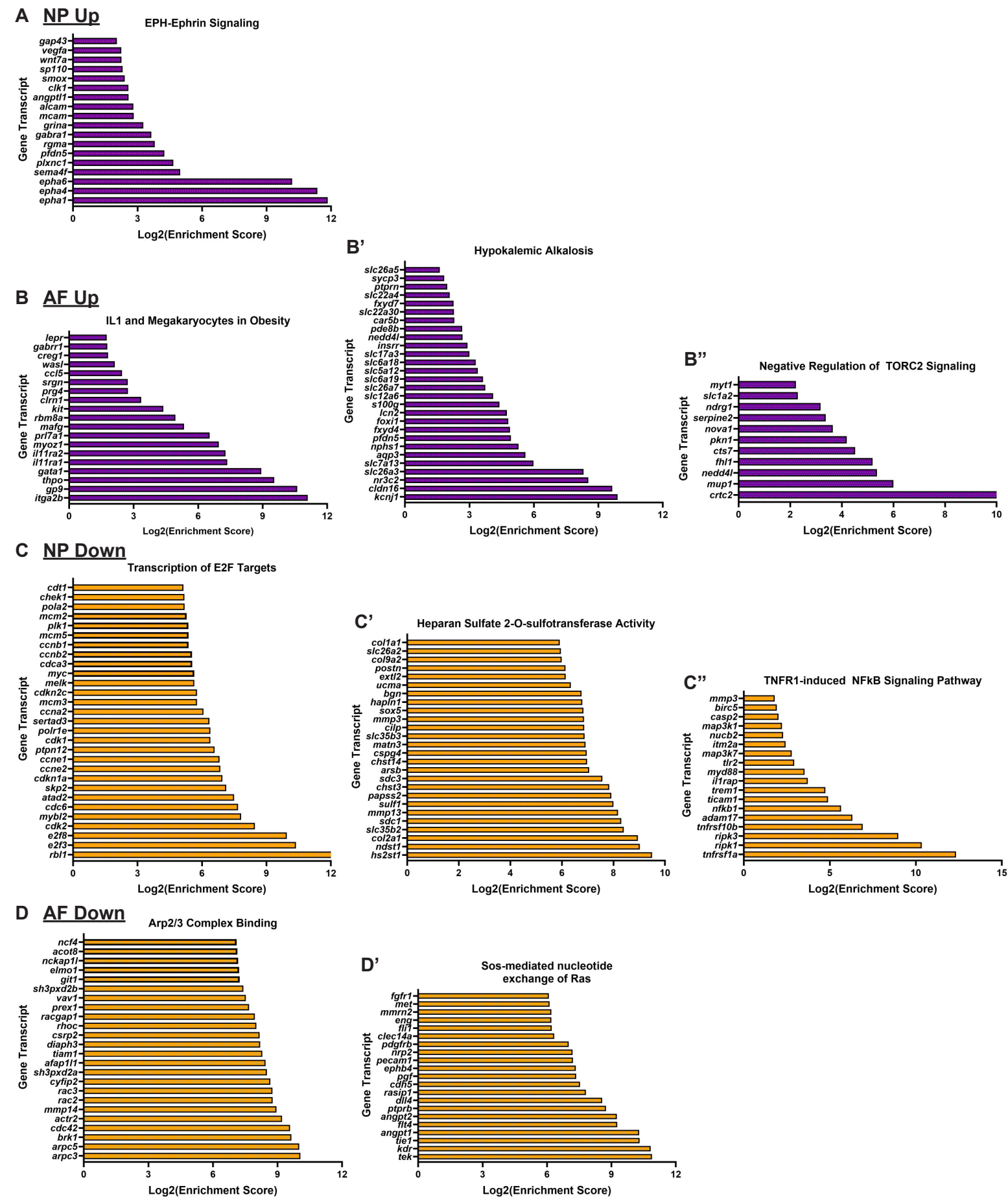

Supplement: Supplementary file 1 — Supplementary Figure 1 [file 41413_2026_526_MOESM1_ESM.pdf]

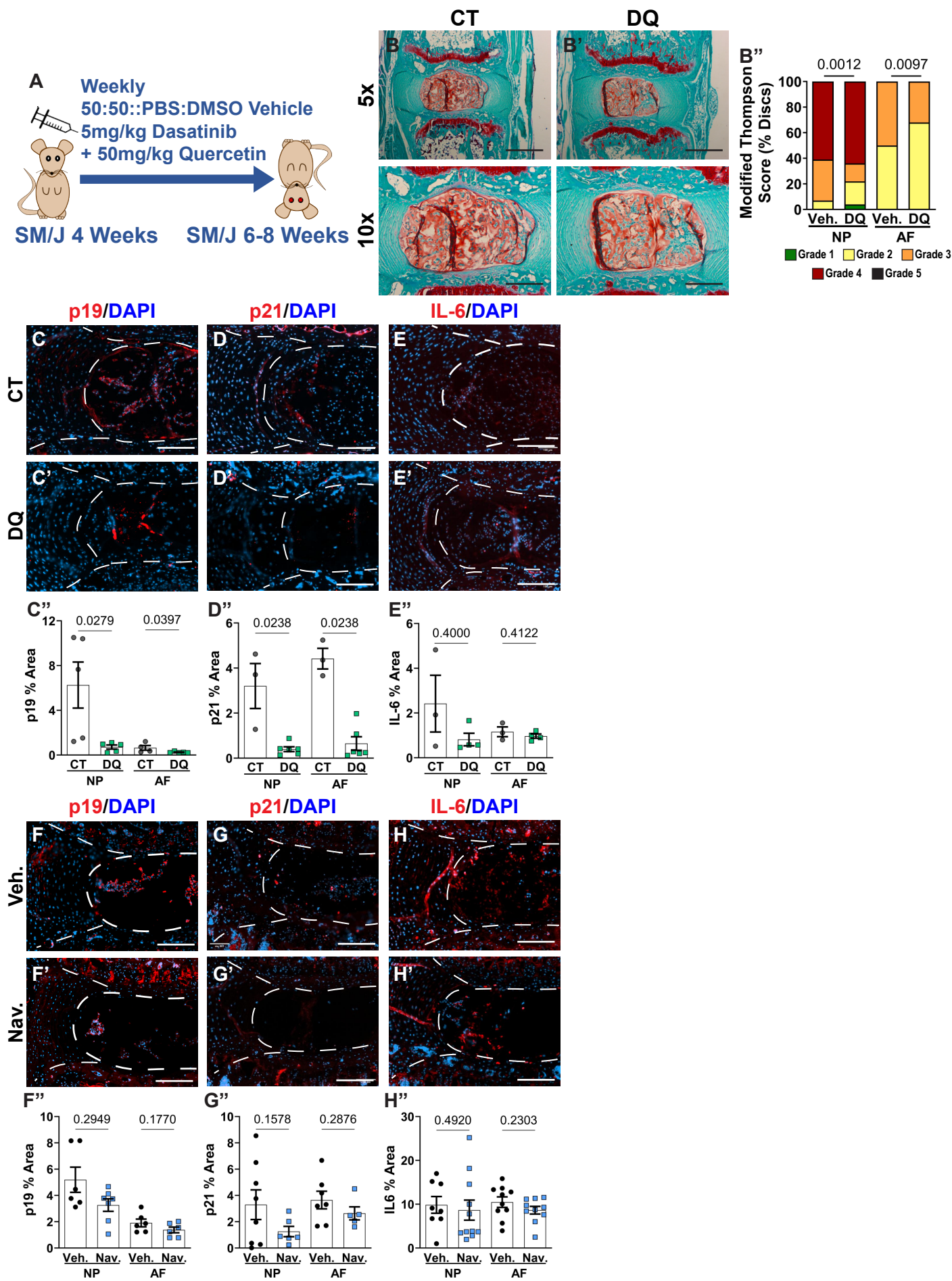

Supplement: Supplementary file 3 — Supplementary Figure 3 [file 41413_2026_526_MOESM3_ESM.pdf]

Supplementary Figure 4

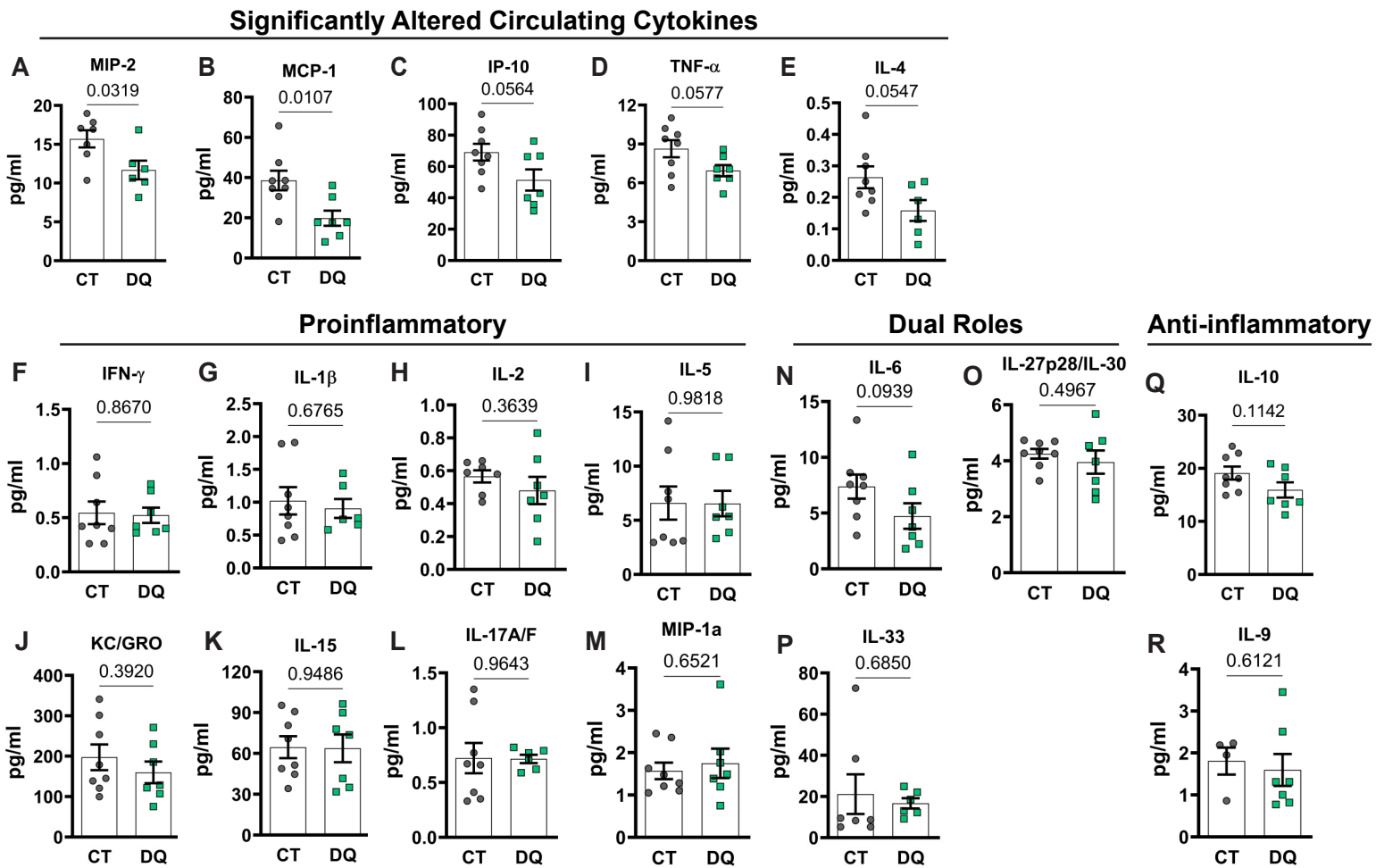

Supplement: Supplementary file 4 — Supplementary Figure 4 [file 41413_2026_526_MOESM4_ESM.pdf]

Supplementary Figure 5

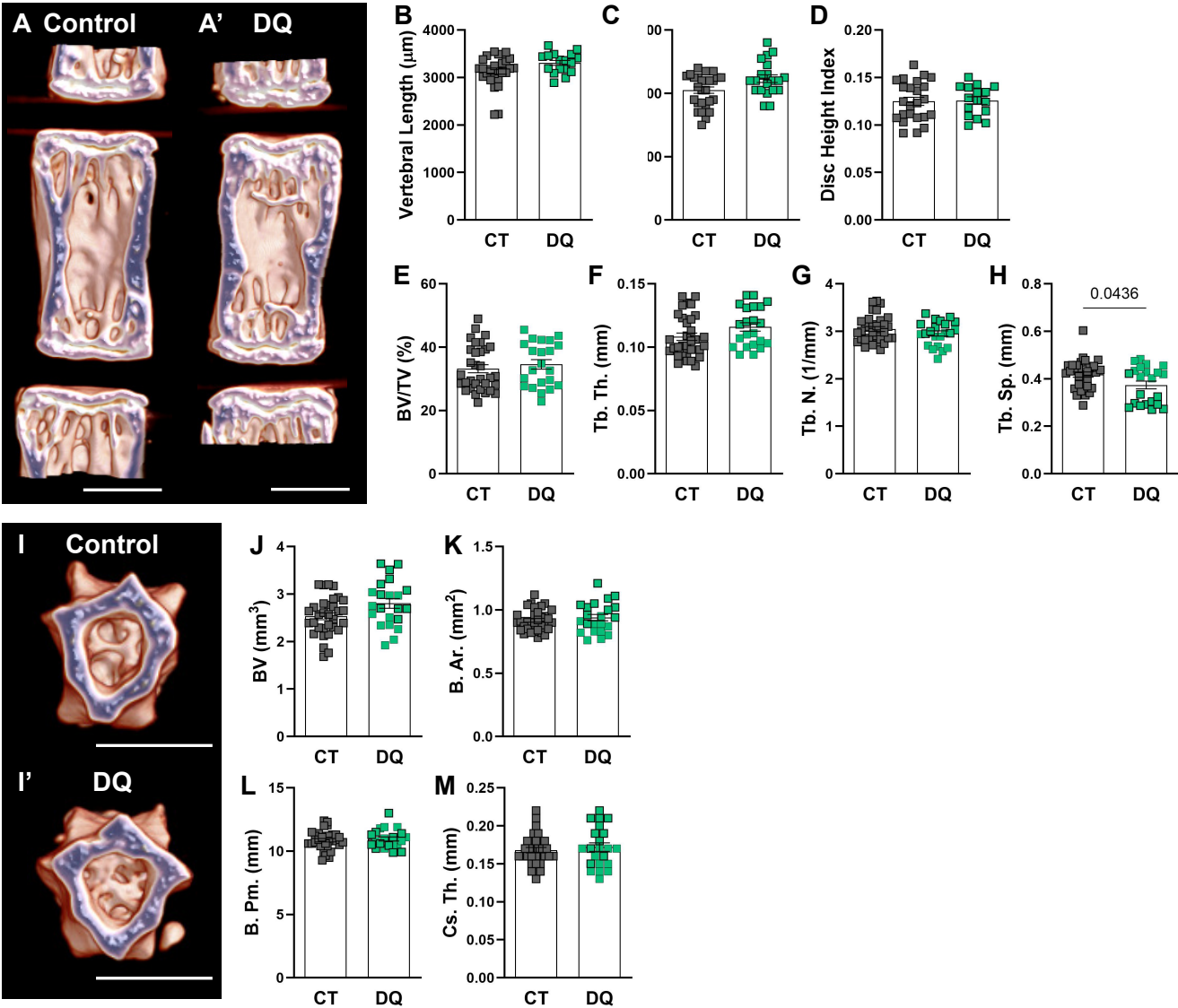

Supplement: Supplementary file 5 — Supplementary Figure 5 [file 41413_2026_526_MOESM5_ESM.pdf]

Supplementary Figure 6

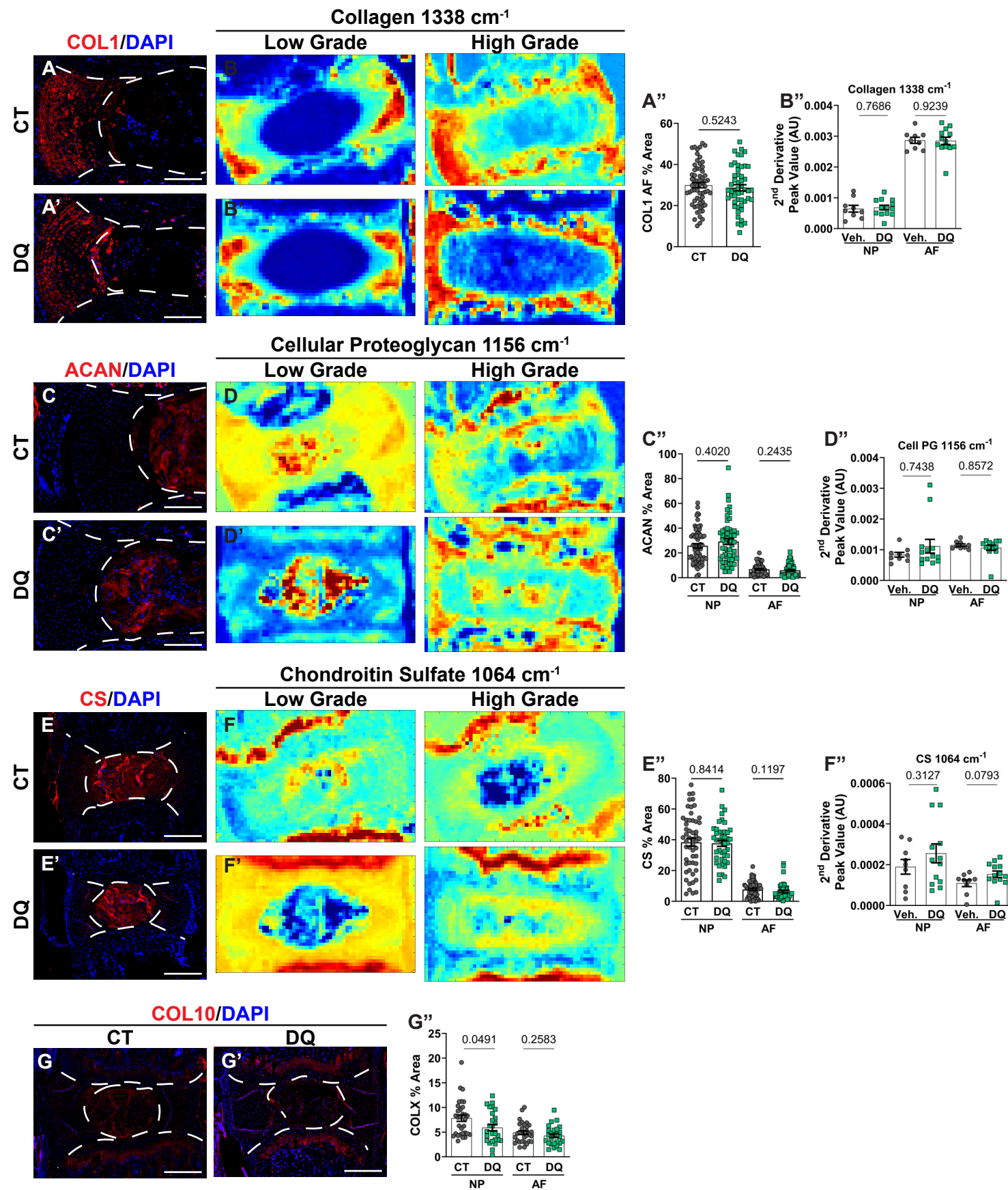

Supplement: Supplementary file 6 — Supplementary Figure 6 [file 41413_2026_526_MOESM6_ESM.pdf]

Supplementary Figure 7

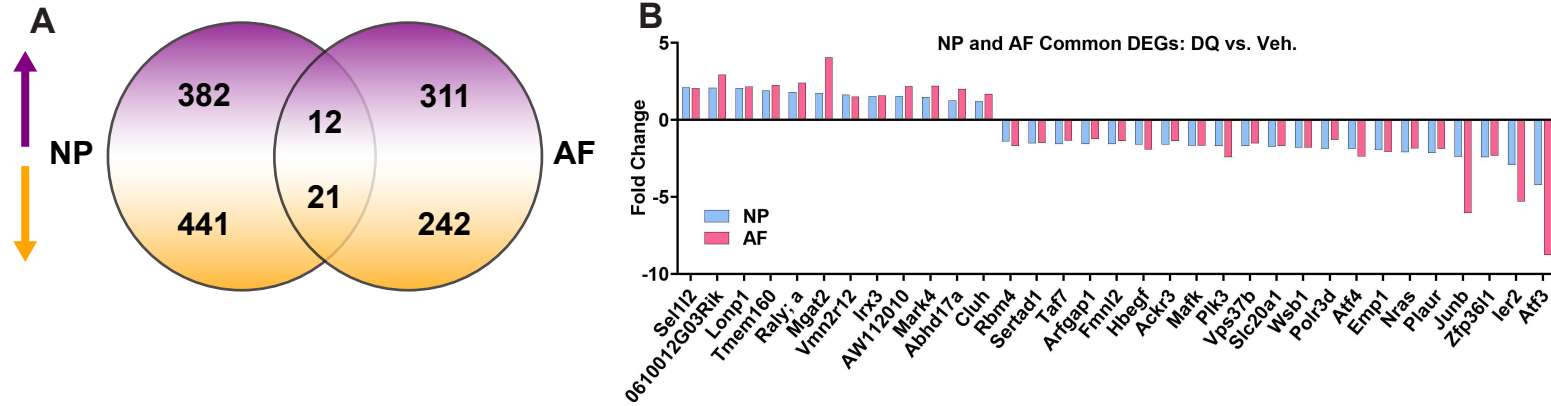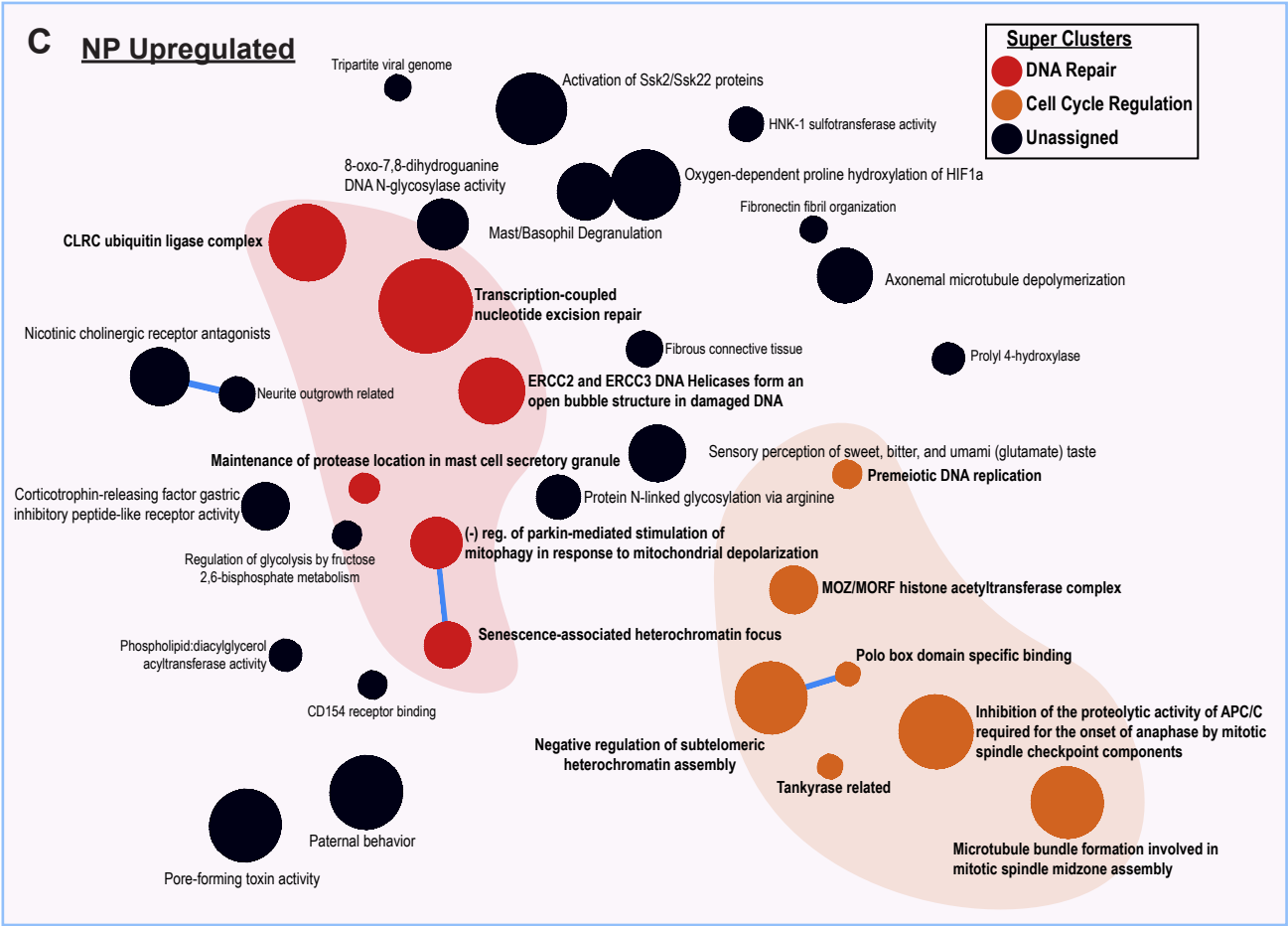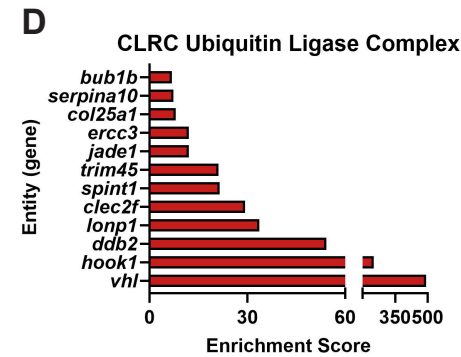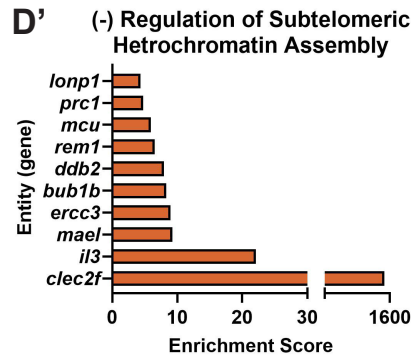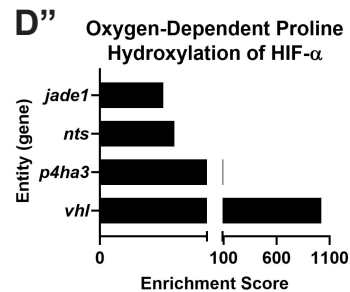

Supplement: Supplementary file 7 — Supplementary Figure 7 [file 41413_2026_526_MOESM7_ESM.pdf]

Supplementary Figure 8

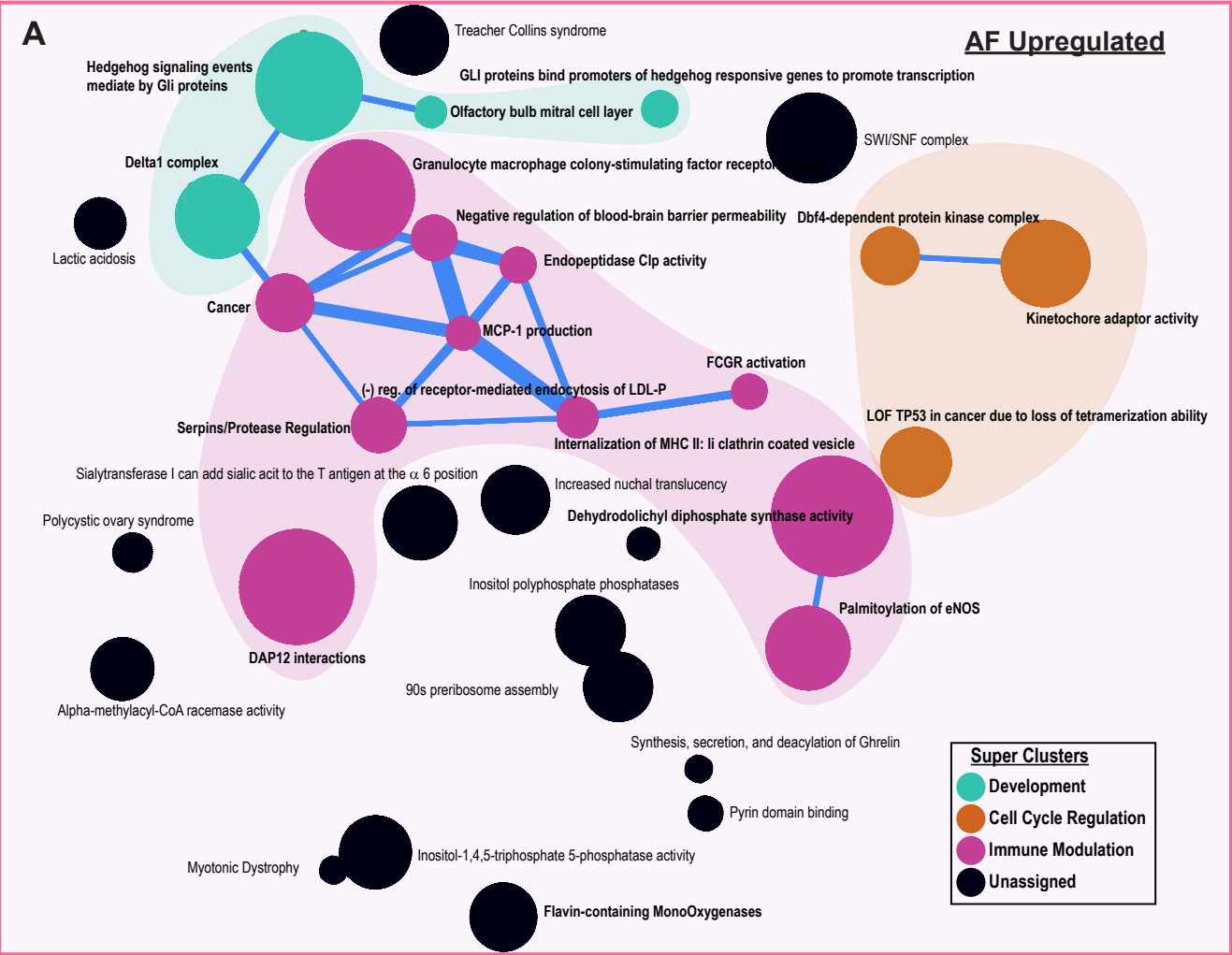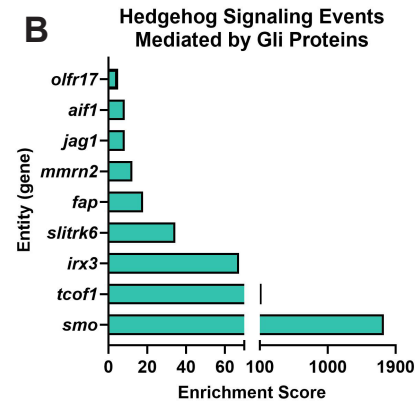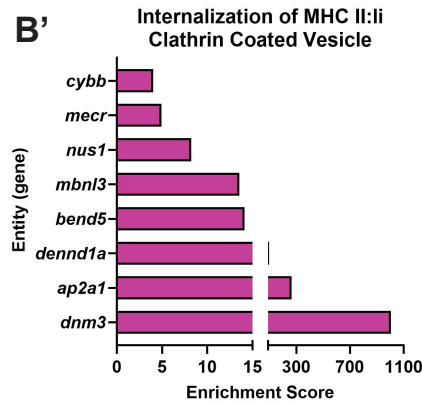

Supplement: Supplementary file 8 — Supplementary Figure 8 [file 41413_2026_526_MOESM8_ESM.pdf]

Supplementary Figure 10

**A**

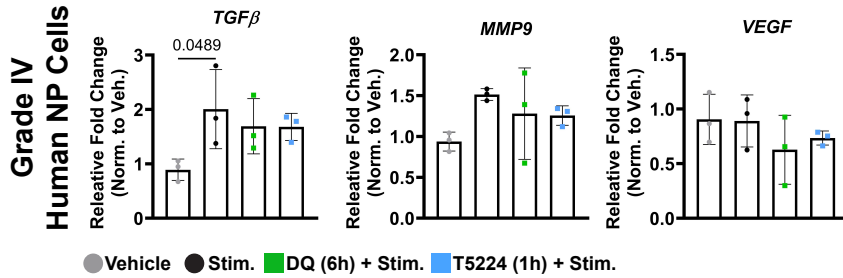

**B**

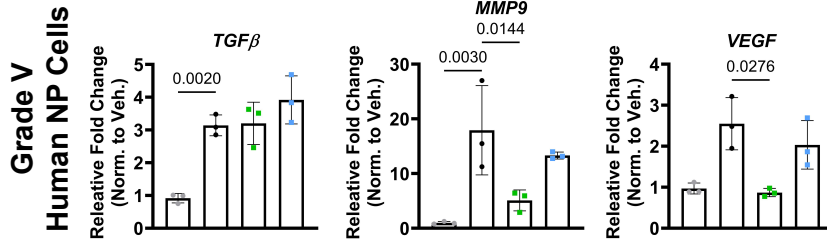

**C**

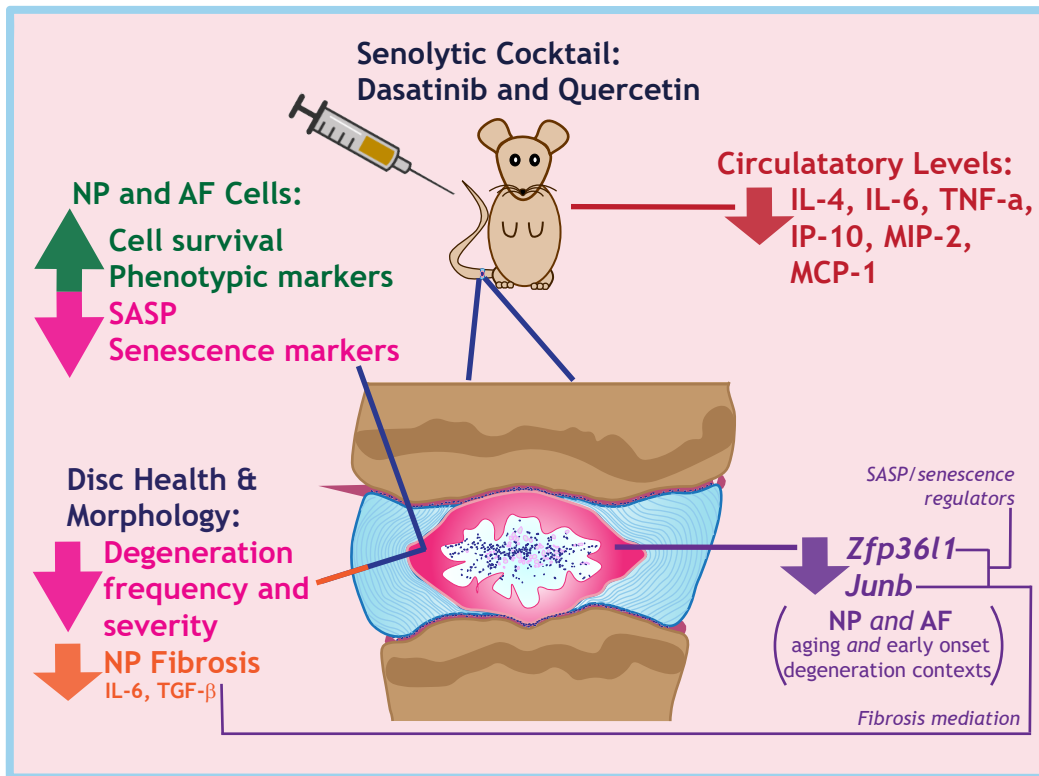

Supplement: Supplementary file 10 — Supplementary Figure 10 [file 41413_2026_526_MOESM10_ESM.pdf]
