# Supplementary material for: Dasatinib and quercetin senolytic treatment delays early onset intervertebral disc degeneration in SM/J mice
Source: Bone Res. 2026 Apr 14;14:42. doi: 10.1038/s41413-026-00526-4 (PMC13076796; doi:10.1038/s41413-026-00526-4)
Supplement: Supplementary file 2 — Supplementary Figure 2 [file 41413_2026_526_MOESM2_ESM.pdf]

Supplementary Figure 2

GLOBAL SSIMILARITY SCORE (ASSERTION ENGINE)					SM/J Model		SPARC Model	B6 Model	
					0.0554;p<0.001	0.0679; p<0.001	0.1193;p<0.001	0.012, P>0.9	0.082, p<0.001
					SMJ_AF_UP	SMJ_NP_UP	SPARC_UP	BL6_AF_UP	BL6_NP_UP
**Theme Name (SenMayo_Murine)**	**ENTITY_TOTAL**	**CONCEPT_TOTAL**	**Pvalue**	**NEScore**	*%overlap*	*%overlap*	*%overlap*	*%overlap*	*%overlap*
**TNF and Lymphotoxin Signaling**	47	**18**	<0.001	3,231	11%	6%	28%	6%	6%
Insulin-like growth factor (IGF) activity regulation by insulin-like growth factor binding proteins (IGFBPs)	39	**9**	<0.001	4,184	22%	11%	22%		22%
**Tissue Inhibitor Of Metalloproteinases (TIMP) associated ECM remodeling**	88	**38**	<0.001	3,609	3%	3%	11%		11%
GP130 Cytokines/Signaling	98	**40**	<0.001	3,361	5%	5%	55%		3%
**VEGF-A/Angiogenesis related**	65	**39**	<0.001	3,243	18%	21%		3%	13%
HSPG2 (perlecan) degradation by MMP3 plasmin (MMP12)	71	**19**	<0.001	3,377	21%	16%	37%		26%
Regulation of systemic arterial blood pressure by capillary fluid shift	75	**37**	<0.001	3,554	5%	14%	16%		3%
Intercellular Adhesion Molecule 1 (ICAM1)-related leukocyte tethering or rolling	93	**36**	<0.001	3,708	3%	3%	31%		6%
**JNK (c-Jun kinases) phosphorylation and activation mediated by activated human TAK1**	87	**39**	<0.001	3,71	3%	3%	13%		5%
**Interleukin-3 Interleukin-5 and GM-CSF signaling**	55	**21**	<0.001	3,792	14%	14%	57%		5%
**Prostaglandin E2 receptor EP2 subtype**	31	**12**	<0.001	3,93	8%		58%	8%	50%
Beta-catenin destruction complex	63	**40**	<0.001	3,974	10%		13%	3%	33%
Placental amniotic mesenchymal stromal cell	53	**37**	<0.001	3,942	8%	38%	5%		3%
Follicular fluid formation in ovarian follicle antrum involved in distinct antral spaces stage	43	**39**	<0.001	3,939	26%	13%	18%		8%
Theme 27	12	**3**	<0.001	3,421	33%	33%		67%	33%
FGFR3b ligand binding and activation	9	**10**	<0.001	3,492	20%	30%	10%		10%
**Galactosylation of collagen propeptide hydroxylysines by procollagen galactosyltransferases**	38	**36**	<0.001	3,532	17%	14%	6%		17%
Eosinophils related in allergic asthma	39	**38**	<0.001	3,585	5%	16%	84%		8%
Alveolar smooth muscle	61	**32**	<0.001	3,364	16%	13%	34%		6%
Inductive mesenchymal to epithelial cell signaling	26	**14**	<0.001	3,336	7%	7%	36%		21%
**Rheumatoid arthritis disease specific synovial fibroblast**	52	**31**	<0.001	3,467	10%	6%	23%		32%
Paroxysmal nocturnal hemoglobinuria (PNH) associated	29	**12**	<0.001	3,503	25%	8%	17%		33%
MyD88-dependent toll-like receptor 2 signaling pathway	52	**20**	<0.001	3,54		10%	50%	5%	20%
Carotid atherosclerotic plaque	42	**40**	<0.001	3,478	5%	5%	13%		23%
**Granulocyte macrophage colony-stimulating factor receptor activity**	78	**40**	<0.001	3,698		8%	55%		3%
Vertebral compression fractures	40	**39**	<0.001	3,901		5%	3%		3%
**Interleukin-2 binding**	47	**11**	<0.001	3,902	9%	27%	36%		
**Th1/Th17 Skewing**	48	**35**	<0.001	3,904	6%	20%	80%		
**CXCR chemokine receptor binding**	43	**4**	<0.001	3,552		25%	50%		25%
**Senescence-Associated Secretory Phenotype (SASP)**	75	**23**	<0.001	3,439	4%		13%		4%
Vasoconstriction of artery involved in ischemic response to lowering of systemic arterial blood pressure	54	**38**	<0.001	3,458		5%	16%		8%
**NGF/TNF (6 C-domain) receptor activity**	56	**11**	<0.001	3,458	9%	9%			27%
Endometrium glandular epithelium	53	**16**	<0.001	3,543	19%	13%	19%		
**PDGF/VEGF Signaling**	63	**6**	<0.001	3,507			33%	17%	17%
Binding of TCF/LEF:CTNNB1 to target gene promoters	19	**9**	<0.001	3,417	11%	11%			
Vascular endothelial growth factor receptor-3 signaling pathway	34	**9**	<0.001	3,796	44%	22%			
**C-X3-C chemokine receptor activity**	69	**5**	<0.001	3,717		20%	20%		
**IL-6-type cytokine receptor ligand interactions**	46	**5**	<0.001	3,603	20%				60%
