# Supplementary material for: Dasatinib and quercetin senolytic treatment delays early onset intervertebral disc degeneration in SM/J mice
Source: Bone Res. 2026 Apr 14;14:42. doi: 10.1038/s41413-026-00526-4 (PMC13076796; doi:10.1038/s41413-026-00526-4)

Supplementary Figure 9

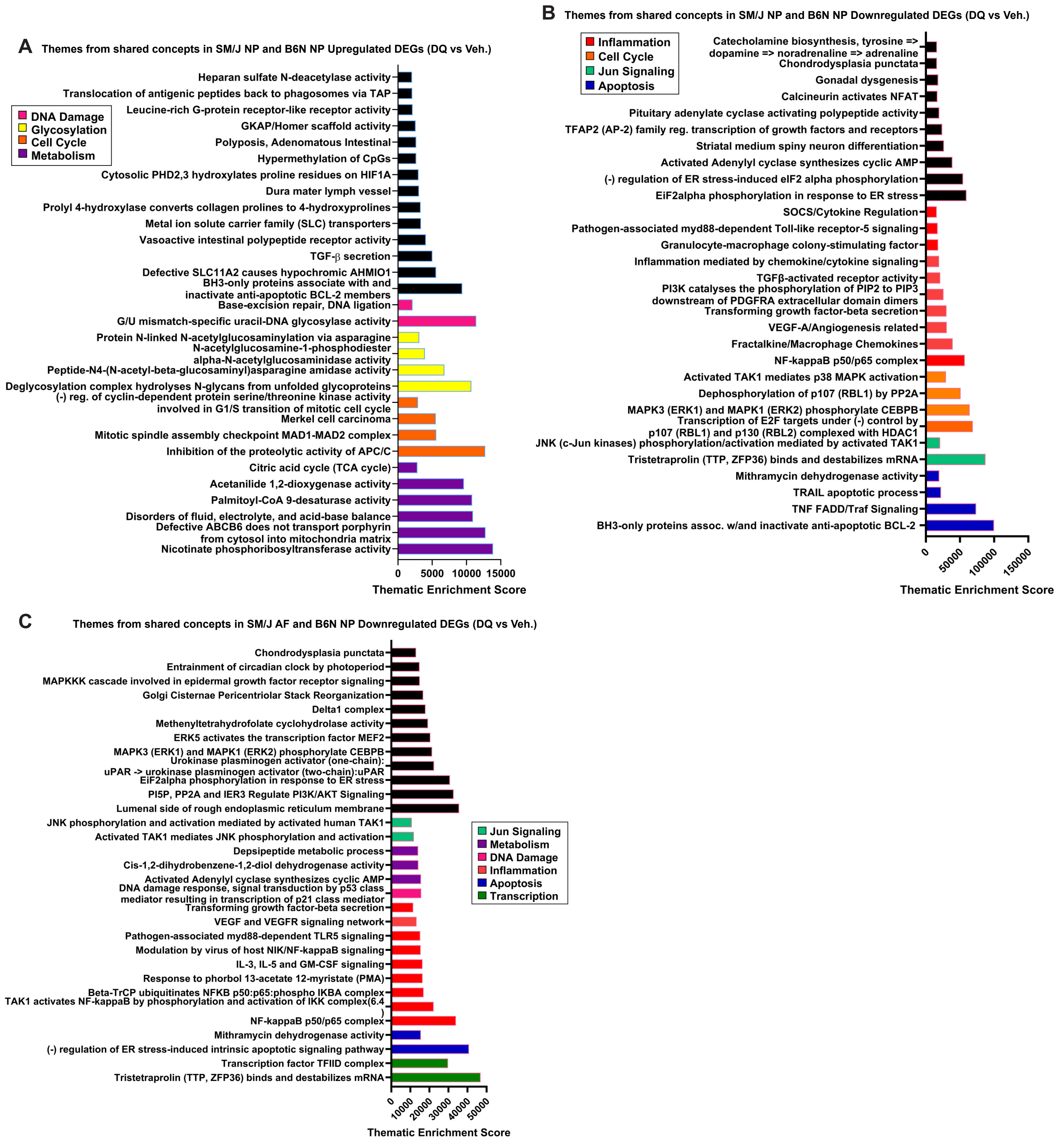

Supplement: Supplementary file 9 — Supplementary Figure 9 [file 41413_2026_526_MOESM9_ESM.pdf]
