# Supplementary material for: Dasatinib and quercetin senolytic treatment delays early onset intervertebral disc degeneration in SM/J mice
Source: Bone Res. 2026 Apr 14;14:42. doi: 10.1038/s41413-026-00526-4 (PMC13076796; doi:10.1038/s41413-026-00526-4)
Supplement: Supplementary file 13 — Supplementary Figures Legends [file 41413_2026_526_MOESM13_ESM.docx]

**Supplementary Figure Legends**

**Supplementary Figure 1. Transcriptomic analysis of 4-week-old and 17-week-old SM/J disc tissues. (A)** *EPH-Ephrin Signaling* is an upregulated theme in the NP **(B)** *IL1 and Megakaryocytes in Obesity* **(B’)** *Hypokalemic Alkalosis* and **(B”)** *Negative Regulation of TORC2 Signaling* are upregulated themes in the AF. **(C)** *Transcription of E2F Targets*, **(C’)** *Heparan Sulfate 2-O-sulfotransferase Activity*, and **(C”)** *TNFR1-induces NFkB Signaling Pathway* are downregulated themes in the NP. **(D)** *Arp2/3 Complex Binding* and **(D’)** *Sos-mediated nucleotide exchange of Ras* are downregulated themes in the AF.

**Supplementary Figure 2. Alignment of SM/J , SPARC, and aged B6 with SenMayo gene set.** Global similarity score (assertion engine) results, including all overlapped themes, concepts, and DEGs between SM/J (4-17wk), SPARC (model of senescence-induced disc degeneration)31, aging B6 (model of age-dependent disc degeneration and senescence)12 tissues, and the SenMayo gene set.

**Supplementary Figure 3. (A)** Schematic showing study design: intraperitoneal injections of DQ or a Vehicle control were administered once every week to mice starting at 4 weeks of age and ending at 6-8 weeks of age. **(B-B’)** Safranin/Fast Green/Hematoxylin staining **(B”)** evaluated with modified Thompson scoring shows DQ improves disc degeneration in SM/J mice after just 2-4 weeks of treatment. Images reflect the range of degenerative outcomes across treatment cohorts. Quantitative immunohistochemistry shows reduced **(C-C”)** p19 (NP and AF) and **(D-D”)** p21 (AF only) in DQ-treated SM/J discs, without altered level of IL-6, **(E-E”) in** 6-8 weeks DQ treated mice. On the other hand, 17-week-old Nv treated mice did not present altered senescent burden in comparison to the Vehicle group **(F-H”).** Distribution statistics were determined using a χ^2^ test. 6-8 weeks old n = 5, (3F, 2M/treatment group). n_Nav._ = 3, n_Veh._ = 3, 2-3 discs levels per animal.

**Supplementary Figure 4.** Multiplex plasma analyses show lower levels of **(H)** MIP-2 and **(I)** MCP-1 in DQ-treated SM/J mice, with downward trends observed in **(J)** IP-10, **(K)** TNF-α, and **(L)** IL-4. Analysis shows no change in **(A-H)** proinflammatory molecules, **(I-K)** cytokines with context-dependent pro- or anti-inflammatory roles, and **(LM)** anti-inflammatory proteins the plasma of SM/J mice receiving DQ treatment. Data are shown as mean ± SD. Significance was determined using an unpaired t-test or Mann-Whitney test, as appropriate.

**Supplementary Figure 5. SM/J vertebral bone is minimally responsive toDQ treatment.** **(A-A’)** Representative μCT reconstructions of the hemi-section caudal vertebrae; **(B)** vertebral length, **(C)** disc height, and **(D)** disc height index are unchanged. Trabecular properties of **(E)** bone volume fraction (BV/TV), **(F)** trabecular thickness (Tb. Th.), and **(G)** trabecular number (Tb. N.) did not change, while there was a mild reduction in **(H)** trabecular separation (Tb. Sp.). **(I-I’)** Representative μCT reconstructions of central cross sections of the caudal vertebrae. Analysis of the cortical properties **(J)** bone volume (BV), **(K)** mean cross-sectional bone area (B. Ar.), **(L)** bone perimeter (B. Pm.), and **(M)** cross sectional thickness (Cs. Th.) were unimpacted by DQ treatment). n_CT_=8 mice (5F, 3M), n_DQ_=6 mice (3F, 3M); 3-5 vertebrae/mouse, 4 discs/mouse. Data are shown as mean ± SD. Significance was determined using an unpaired t-test or Mann-Whitney test, as appropriate.

**Supplementary Figure 6. Immunohistochemistry and FTIR analysis show comparable extracellular matrix profiles between DQ- and Veh.-treated 17-week-old SM/J mice.** Quantitative immunohistological staining shows no change in **(A-A”)** COL1, **(C-C”)** ACAN, or **(E-E”)** chondroitin sulfate (CS) abundance in DQ-treated mice. **(G-G”)** COL10 abundance was significantly reduced in the NP by DQ treatment. Data are shown as mean ± SD. Significance was determined using an unpaired t-test or Mann-Whitney test, as appropriate. Distribution statistics were determined using a χ^2^ test. n_DQ_ = 5-7, n_CT_ = 5-7, 3-4 levels each animal. **(B-B”)** Chemical map of mean second-derivative peaks for collagen and quantification of mean second-derivative peaks for collagen (1338 cm^−1^). **(D-D”)** Chemical map of mean second-derivative peaks and quantification of mean second-derivative peaks for proteoglycan (1156 cm^−1^). **(F-F”)** Chemical map of mean second-derivative peaks and quantification of mean second-derivative peaks for chondroitin sulfate (1064 cm^−1^). Red: high relative absorbance; yellow: intermediate relative absorbance; blue: low relative absorbance. Quantification of mean second-derivative peaks for collagen (1338 cm −1). n_DQ_ = 5-7, n_CT_ = 5-7, 3-4 levels each animal.

**Supplementary Figure 7.** **(A)** 859 DEGs were identified in the NP, and 586 DEGs were identifies in the AF, with 33 transcripts commonly mediated by DQ in SM/J mice. **(B)** Commonly differentially expressed genes in NP and AF tissues of DQ-treated SM/J mice. **(C-D”)** Compbio analysis highlighted themes relating to DNA repair (red) and cell cycle (orange) among upregulated DEGs in the NP. 4-week-old (n = 6), 17-week-old CT and DQ mice (n = 6 mice/treatment).

**Supplementary Figure 8. (A-B’)** Compbio analysis of AF tissues highlighted themes relating to development (turquoise), cell cycle (orange), and inflammatory signaling (pink) among upregulated DEGs. 17-week-old CT and DQ mice (n = 6 mice/treatment).

**Supplementary Figure 9. (A)** Significant themes upregulated by DQ in SM/J NP and B6N NP **(B)** significant themes downregulated by DQ in SM/J NP and B6N NP **(C)** significant themes downregulated by DQ in SMJ AF and B6N NP.

**Supplementary Figure 10. (A)** Grade IV Human NP cells exhibit lower expression of *TGFβ* compared to the stimulus group and no changes in *MMP9* and *VEGF*. **(B)** Grade V human NP cells exhibit lower expression of *TGFβ* compared to the stimulus group. Additionally, DQ treatment resulted in a decrease in *MMP9* and *VEGF*. Data are shown as mean ± SD. Significance was determined using a Dunnett's multiple comparisons test (n = 3 independent experiments, performed in triplicate). **(C)** Schematic summarizing the improved degenerative outcomes of SM/J discs treated with DQ: SM/J mice treated with DQ from 4 weeks of age to 17 weeks of age exhibit a reduced frequency and severity of intervertebral disc degeneration, characterized by a decrease in degenerative scores and reductions in fibrosis and fibrotic markers. This was associated with improved disc cell survival, a retention of disc phenotypic markers, and a reduction in senescence and SASP markers. DQ treatment also alleviated systemic inflammation in SM/J mice. Transcriptomic analysis revealed *Zfp36l* and *Junb* as potential regulators of these processes, as they are known to be upstream of master senescence regulators and involved in the regulation of IL-6 and TGF-β.
